# Genome Sequences of Cluster E Mycobacteriophages Xandras and BigBubba

**DOI:** 10.17912/micropub.biology.001460

**Published:** 2025-03-13

**Authors:** Ashley Gledhill, Gabriel S. Gooden, Janelle Aguazul, Aanyaa Arora, Ava R. Blackledge, Michael H. Bucks, Tessa Carby, Andrew Cu, Jacob Fulkerson, Chealsea W. Gachagua, Elly Grogan, Haley H. Hanson, Gillian N. Johnston, Lola S. Norman, Lindsey M. Oak, Grant J. Oller, Het Parekh, Sakshi Patel, Sydney Putnam, Grant Spalding, Jacob Thomas, Patrick Wallace, Claire A. Rinehart, Rodney A. King

**Affiliations:** 1 Biology Department, Western Kentucky University, Bowling Green, Kentucky, United States

## Abstract

Bacteriophages Xandras and BigBubba were isolated on *Mycobacterium smegmatis* mc ^2^ 155 from enriched soil samples. Xandras' genome length is 75,179 bp with 144 predicted protein-coding genes and two tRNAs. BigBubba's genome length is 75,006 bp with 147 predicted protein-coding genes and two tRNAs. Each genome contains a cyclic oligonucleotide sequestering protein (CBASS antagonist).

**Figure 1. Transmission electron microscopy images of phages Xandras and BigBubba:**
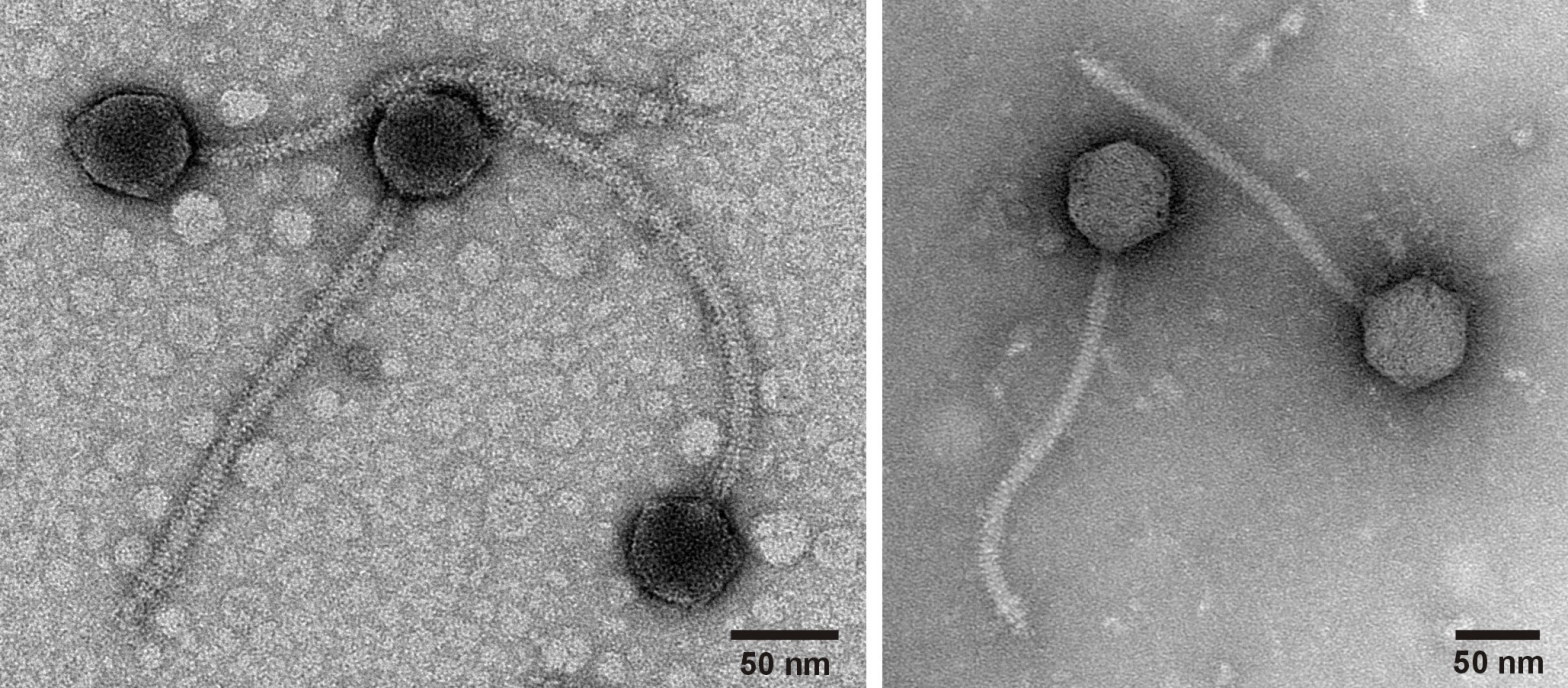
Negative stain (1% uranyl acetate) transmission electron microscopy images of Xandras (left) and BigBubba (right) shows a siphovirus morphology with an icosahedral capsid and a flexible tail. See text for particle dimensions.

## Description

Bacteriophages are highly diverse and abundant viruses that infect bacteria and have been used as therapeutic agents to fight antibiotic resistant bacterial infections (Hatfull et al., 2022). The genomes of two double stranded DNA (dsDNA)-tailed bacteriophages infecting *Mycobacterium smegmatis* mc ^2^ 155, Xandras and BigBubba, are reported here. Phage isolation and genome analysis were conducted at Western Kentucky University as part of the Howard Hughes Medical Institute's Science Education Alliance Phage Hunters Advancing Genomics and Evolutionary Science (HHMI SEA-PHAGES) program (Jordan et al., 2014). Xandras and BigBubba were isolated using standard enrichment techniques (Zorawik et al., 2024). Briefly, soil samples were resuspended in Middlebrook 7H9 liquid medium, inoculated with *M. smegmatis* mc ^2^ 155, and incubated with shaking at 30˚C. After 48 hours, the cultures were centrifuged, the supernatants were filtered (0.2 um pore filter), the filtrates plated in top agar with *M. smegmatis,* and plates incubated at 30˚C, yielding plaques with clear centers and turbid halos with a diameter of ~ 0.5 cm after 48h. Both phages were purified through three rounds of plating.

Electron microscopy on negatively stained (uranyl acetate, 1%) particles revealed that Xandras and BigBubba are siphoviruses with average head diameters of 57nm (SD=6nm) and 64nm (SD=4nm) and non-contractile tails averaging 244nm (SD=11nm) and 263nm (SD=16nm), respectively (n=10). Phage genomic DNA was extracted from a lysate using the Promega Wizard Cleanup Kit, prepared for sequencing using the NEB Ultra FSII kit, and sequenced via the shotgun Illumina sequencing method (v3 reagents), resulting in 551,532 and 385,212 150 base single end reads for Xandras and BigBubba, respectively. Reads were assembled with Newbler v2.9 to generate contigs with 1,039-fold (Xandras) and 728 fold (BigBubba) coverage, and the genomes checked for completeness and termini type using Consed v29 (Gordon and Green, 2013; Russell and Hatfull, 2018). The completed genomes of Xandras and BigBubba are 75,179 bp and 75,006 bp, respectively, and they share the same 9-base 3' single-stranded overhang sequence (5' -CGCTTGTCA- 3'). The G+C content of Xandras and BigBubba is 62.9% vs 62.8%, respectively, only slightly lower than the bacterial host, *Mycobacterium smegmatis* mc ^2^ 155 (67.4%).

Genes were predicted using Glimmer (Delcher et al., 2007), Genemark (Besemer and Borodovsky, 2005), Aragorn (Laslett and Canback, 2004), tRNAscan-SE (Lowe and Eddy, 1997), and manual inspection and revision using PECAAN (discover.kbrinsgd.org). Within PECAAN, gene functions were assigned using HHPRED (Söding et al., 2005) alignment to the PDB_mmCIF70, Pfam- v.36, NCBI Conserved Domains databases, and BLAST (Altschul et al., 1990), alignment to the NCBI nonredundant protein (National Center for Biotechnology Information) and PhagesDB (Russell and Hatfull, 2016) databases. Default settings were used for all software. Xandras has 144 predicted protein-coding genes and two tRNA genes whereas BigBubba contains 147 predicted genes and two tRNAs. Based on gene content similarity of at least 35% to phages in the Actinobacteriophage database, phagesDB, both Xandras and BigBubba are assigned to actinobacteriophage cluster E (Pope et al, 2017; Russell and Hatfull, 2016).

Functions were predicted for approximately 38% of the called genes. Structural proteins (e.g. major capsid and tail proteins) are located in the left region of the genomes, followed by the lysis cassette which includes the lysin A, holin, and lysin B genes. Xandras and BigBubba both contain an immunity cassette that includes a tyrosine integrase and the immunity repressor and are thus predicted to be temperate phages. Predicted DNA metabolism and replication genes are located on the right arm of the genome. Roughly a quarter of all Xandras and BigBubba genes are transcribed in the reverse direction and most have no known function.

Xandras and BigBubba are unusual because they encode cyclic nucleotide sequestering proteins (CBASS antagonist) (Huiting et al., 2023); genes 101 and 102, respectively. Triggered by phage infection, cGAS-like enzymes produce cyclic oligonucleotides which serve as signaling molecules and initiate anti-phage responses. Phages have evolved anti-CBASS proteins, like Acb2, to counter this bacterial defense. Acb2 binds to cyclic oligonucleotides, hindering their activity and impeding the bacterial immune response (Huiting et al., 2023). The role this plays in mycobacteriophage infections is unknown.

**Annotation and sequencing accession**

**Table:** 

Phage	Genbank Accession	SRA Accession	Number of reads
Xandras	PP750962	SRX26785854	551,532
BigBubba	PP750964	SRX26785847	385,212
